# Machine-Readable Structural Information Is Essential for Natural Products Research

**DOI:** 10.1021/acs.jnatprod.5c00836

**Published:** 2025-10-20

**Authors:** Karin Steffen, Nicholas H. Oberlies, Antonis Rokas

**Affiliations:** † Department of Biological Sciences and Evolutionary Studies Initiative, 5718 Vanderbilt University, Nashville, Tennessee 37235, United States; ‡ Department of Chemistry and Biochemistry, 14616 University of North Carolina at Greensboro, Greensboro, North Carolino 27402, United States

**Keywords:** data accessibility, chemical structures, digitization, SMILES, InChI, InChIKey, MOLfile, chemoinformatics, cheminformatics

## Abstract

Structural drawings of secondary metabolites encapsulate a wealth of information, but their static nature hinders their sharing and reuse. At a time when research increasingly relies on, and benefits from, access to comprehensive natural products “big data”, this hindrance is contrary to the norms of science. This Perspective discusses current challenges in sharing and accessing structure data and offers a simple solution, namely, publishing machine-readable text descriptors for new secondary metabolites. Requiring such descriptors will be straightforward to implement for both authors and publishers and will help derive even greater value from the efforts expended to understand the chemistry of Nature.

## Graphical abstract

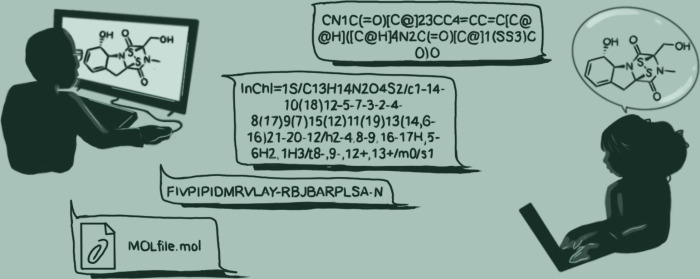

## Introduction

Natural products chemistry is a hugely valuable field that informs agricultural,^1,2^ biological,^3,4^ clinical,^5,6^ ecological,^7^ evolutionary,^8,9^ and pharmaceutical^10,11^ research.^12^ As such, our field attracts scientists with a variety of backgrounds, including many outside of chemistry.

A foundational aspect of natural products chemistry is the discovery and description of new chemical compounds, often termed secondary metabolites or specialized metabolites.^13,14^ To date, an estimated 400,000 secondary metabolites have been described from Nature.^15,16^ However, digital access to this wealth of structures remains challenging.^17,18^ To borrow an example from our own research on fungi, identifying and generating structural drawings for secondary metabolites known to be produced by a fungal species or group—say, *Aspergillus fischeri* or the genus *Aspergillus*—is nontrivial even though this is arguably one of the best-studied fungal taxa with respect to secondary metabolism.^19–21^

This lack of digital access is not for the lack of effort on the computational side.^22^ A recent review listed over 120 databases and collections of compounds from Nature published since 2000, 98 of which were still accessible in 2020.^17^ Although few of these are open-access or allow one to freely mine and download the data,^17^ arguably the main obstacle for the curation and maintenance of compound repositories is the lack of digitization of the structures of both known and new compounds.^18^

In this Perspective, we advocate for a change in practices that address this challenge head on, namely, the provision of machine-readable structural descriptors when publishing natural product structures. We begin by briefly outlining the value and importance of chemical structures and then highlight limitations for reusing those structures imposed by the current publication standards. We next argue how the provision of machine-readable structural information—a small and readily implementable change in publishing practices—will facilitate automated deposition and retrieval, reduce structural errors across databases and the literature, and allow for data access according to FAIR (Findability, Accessibility, Interoperability, and Reusability) principles, democratizing natural products chemistry in the ”big data” era.^23^

## The Structures of Secondary Metabolites Have Vast Potential for Use after Their Publication

Individual compounds from Nature frequently serve as inspirations or templates for analogue and total synthesis.^24^ Additionally, larger collections of natural product structures find many applications in analytical chemistry and metabolomics, for example, by enhancing the capabilities of molecular networking structure annotations via global natural products social molecular networking (GNPS)^25^ or when elucidating unknown structures by mass spectrometry or NMR. Chemical structures can be used to calculate and infer many properties of a molecule. These include various fundamental physicochemical properties, such as molecular volume, molecular weight, charge, hydrogen bond acceptors or donors, the acid dissociation constant (p*K*_a_), or the partition coefficient (LogP), to name just a few. Large-scale analyses of natural product structures in repositories have been used for decades in cheminformatics, aiding in estimating properties instead of explicitly measuring them, e.g., predicting toxicity to reduce animal testing,^26^ virtual screening to identify potential drug leads,^27^ predicting bioactivities,^28^ and predicting biosynthetic pathways.^19^ For example, many studies identifying the biosynthetic origin (i.e., the biosynthetic gene cluster, BGC) of a secondary metabolite (SM) do so by using structurally similar compounds and their corresponding BGCs as a guide for discovery.^29–31^ Such BGC-SM data are currently manually collected in crowdsourced efforts in the open access database MIBiG.^32^ In addition, the emerging applications of AlphaFold, artificial intelligence, and machine learning approaches to natural products research hold vast potential for novel discoveries, but their performance crucially depends on large amounts of well-curated training data.^33,34^ Hence, for these and many more applications and use cases of natural products, the accuracy of secondary metabolite structures is pivotal for achieving reliable outcomes.

## Errors Can Arise When Static Drawings of Secondary Metabolites Are Relied Upon

A chemical structure depicts the molecular geometry, i.e., the spatial arrangement of atoms, in a molecule as well as bond types and charges when available and necessary. Modern chemical structures are credited to August Kekulé and Archibald Scott Couper, who independently proposed graphical representations of atoms in molecules connected by bonds over 160 years ago.^35,36^ Since then, many types of structural formulas have been developed and are in concurrent use, including chair conformations, wedge-dash diagrams, Lewis, Markush and skeletal structures, and Newman, Haworth, or Fischer projections, just to name a few.^37,38^ Yet, all these chemical representations remain graphical and potentially complex, since secondary metabolites, in particular, are frequently characterized by diverse scaffolds featuring multiple stereogenic centers and complex ring systems.^39^

Chemical structure drawings, while very informative, create an inherent challenge in current publishing practice by hindering the straightforward computational search, sharing, and reuse of information.^22^ Therefore, anyone who wishes to reuse chemical structure drawings of secondary metabolites has three options: redrawing the molecule(s) with chemistry software, extracting the information with the help of emerging AI computer vision tools such as DECIMER.ai/MARCUS,^22,40,41^ or relying on the presence of the secondary metabolite(s) in a database. These options lead to two major consequences:

(1) **Reproduction introduces and propagates errors**. Errors can arise from incorrect automated extraction of the secondary metabolite structure itself and/or the connection of structure and trivial name, from human error when redrawing, and/or from mistakes when data were deposited in a database (Figure 1).^22,26,42,43^ Reasons for those errors include the variation in the level of literacy in reading, understanding, interpreting, and applying structural drawings of molecules between scientists.^44,45^ The coexistence of various guidelines and recommendations for depicting structures, together with personal preference or norms for various subdisciplines, further complicate the interpretation, collection and reuse of structure representations.^42,46,43^ For example, it is common in the organic chemistry community to represent side chains with shorthand abbreviations (e.g., “Boc” for *tert*-butyloxycarbonyl), whereas natural products chemists may draw out the side chains or use abbreviations that are common to their particular subspecialty (e.g., “tiglyl” for a group derived from tiglic acid, *E*-2-methylbut-2-enoyl). In short, errors in reinterpreting structures happen and are often propagated unintentionally.
(2) **Current publishing practices hinder automated and accurate correction or addition of known and new secondary metabolites structures in databases**. This severely limits the generation of freely available, comprehensive, accurate and up-to-date databases of structures of compounds derived from Nature.^17,18,43^ This fact is illustrated by the many collections of secondary metabolite structures published in the last two decades being (virtually) static,^17^ as the effort to manually populate them is too high. At the same time, the very large number of structural databases highlights both the need for large-scale accessible data and the lack of a generally accepted solution that addresses that need.

**Figure 1:**
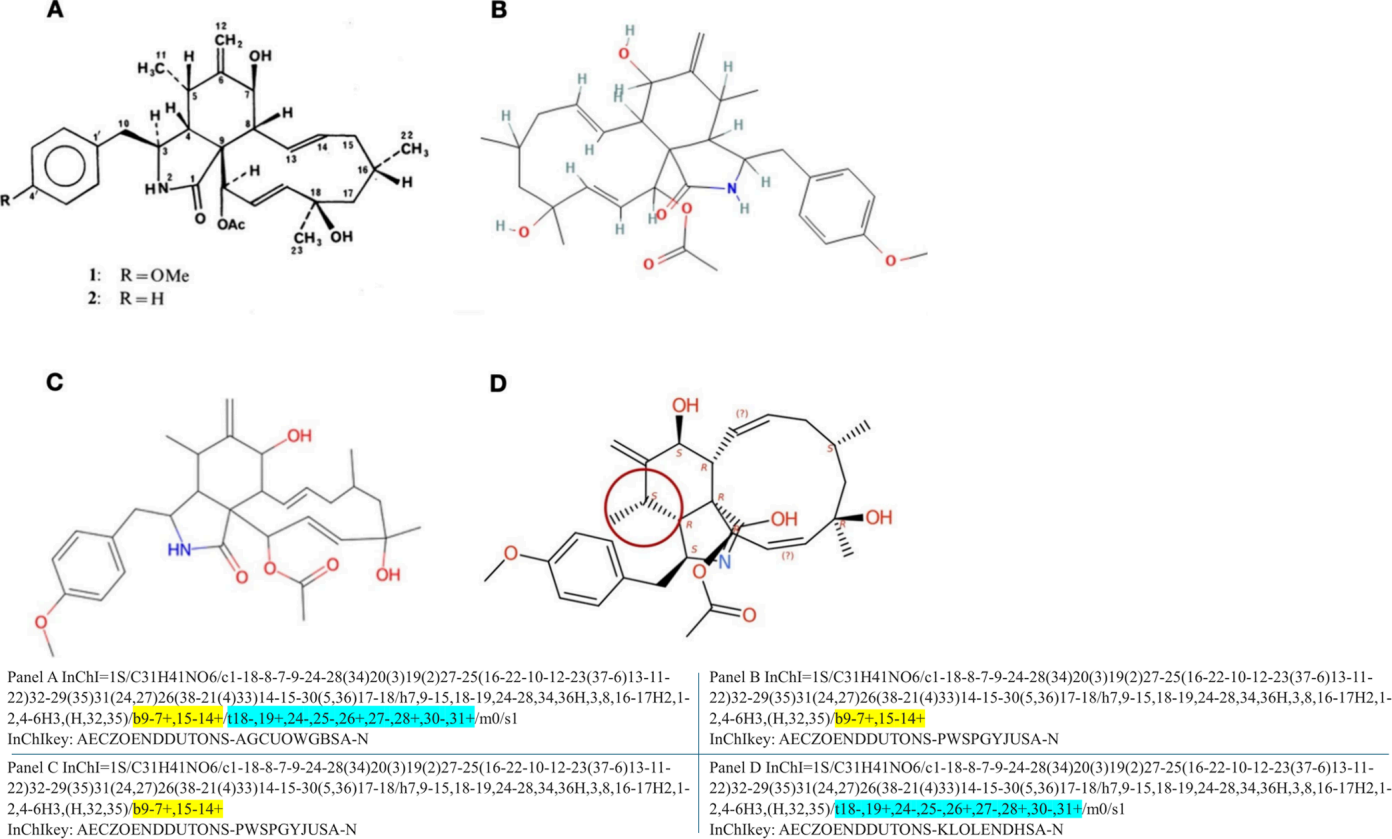
Four different publicly available structural drawings of the fungal secondary metabolite pyrichalasin H (**1**). (A) The original structure as published by Nukina.^47^ (B) Structure in the PubChem database (accession number CID6442793). (C) Structure in the NPAtlas database (accession number NPA028640). (D) Structure in the COCONUT database (accession number CNP0376537.1). The circled area of structure D illustrates where a rule of organic chemistry is being broken; a carbon atom should not have a down wedge stereodescriptor bond going both to it and away from it. The InChIs and InChIKeys for the four structures are given above. The blue highlights show where the absolute configuration is notated, and the yellow highlights show where the double bond geometry is notated.

Overall, current publishing practices slow down progress and limit discovery by requiring researchers to devote considerable (and we would argue unnecessary) effort to redrawing structures, e.g., for generating data sets, for digital lab books, or for new publications. The same practices also hinder large scale investigations dependent on access to comprehensive data on secondary metabolite structures.

## To Efficiently Make Use of Secondary Metabolite Structures, Machine-Readable Structure Representations Are Essential

There is a simple and straightforward solution to maximize the potential for discovery and alleviate the inevitable errors that accumulate when only static drawings of chemical structures are reported: publishing the digital, i.e., machine-readable, structural information for each compound. There are several ways of doing so using line notations such as SMILES (Simplified Molecular Input Line Entry System)^48^ or InChI (International Chemical Identifier)^49,50^ (Figure 2) or files with 3D information such as MOLfiles (SDF files). Ideally, one would also provide the InChIKey hash together with these machine-readable structures for authenticating reproductions of a structure.^49,50^ Subsequently, we describe these chemical data formats and discuss the differences, advantages, and limitations for each of them. An example with all chemical data formats and their reproducibility is given in Figure 3.

**Figure 2:**
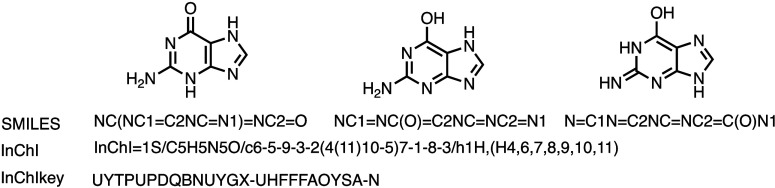
Guanine has a total of 16 tautomers,^43^ each with a different SMILES descriptor derived from the individual structural representation of the molecule. Three examples are shown above. In contrast, they all have identical InChI and InChIKey descriptors, as all tautomers have the same connectivity. Thus, while SMILES descriptors may seem more intuitive or exact for a given structure, they have the potential to linger as duplicate entries in databases because different SMILES descriptors from the same structure may not be automatically matched up. In turn, in cases where bond types matter, SMILES or MOLfiles could be preferred over InChIs for their explicit definition of bond positions.

Both SMILES and InChI descriptors are line notations, text strings of letters and symbols computed from structures. They have a variable length, depending on the structure they encode. As text strings, SMILES and InChI line notations allow copy/pasting, sharing, and reusing of structural information. Crucially, the structure of the molecule can be regenerated from both SMILES and InChI descriptors via cheminformatic software (e.g., ChemDraw, RDkit^51^) and other widely used software with reservation for variation due to preferred orientation of display not defined in the line notations.

SMILES were developed by Daylight chemical information systems and others over time.^48,52^ They have a foundation in graph theory and represent structural information in a highly condensed manner. A SMILES recapitulates each atom and bond in a human-readable way. However, there are two related features of SMILES that can lead to confusion. First, one structure can have many valid SMILES. An intuitive example of several SMILES existing for the same molecule is tautomers (Figure 2), but other examples exist, e.g., when ring structures are broken in an arbitrary position. Second, there are several different types of SMILES, such as generic, unique, canonical, isomeric, and absolute SMILES.^52,53^ Importantly, all but one of the different types of SMILES only contain 2D information. The sole exception is the isomeric (including “absolute”) SMILES type, which contains 3D information with the symbols “@”, “/”, and “\” indicating configurations at chiral centers or double bonds.

InChI, developed by IUPAC, is a single format with occasional updates.^50^ Due to the chemical logic applied when computing an InChI from a structure, it is more abstract than a SMILES. One explicit design goal or feature of an InChI is that one molecule (including its tautomers) is represented by a single InChI (Figure 2). The InChI string starts with “InChI = 1/” and is composed of several layers of information separated by “/”. The first layer is the chemical sum formula, followed by skeletal connectivity “/c”, hydrogen layer “/h”, and others, with some layers being optional.^49^

SMILES and InChI descriptors can be converted into each other, but they are not universally interoperable. One can be calculated from the other, but due to differences in how information is stored in both line notations, some information can be lost in the conversion process, e.g., pertaining to the exact position of single/double bonds in structural isomers, as when generating InChIs that information may be removed or changed (see also Figure 3).

**Figure 3:**
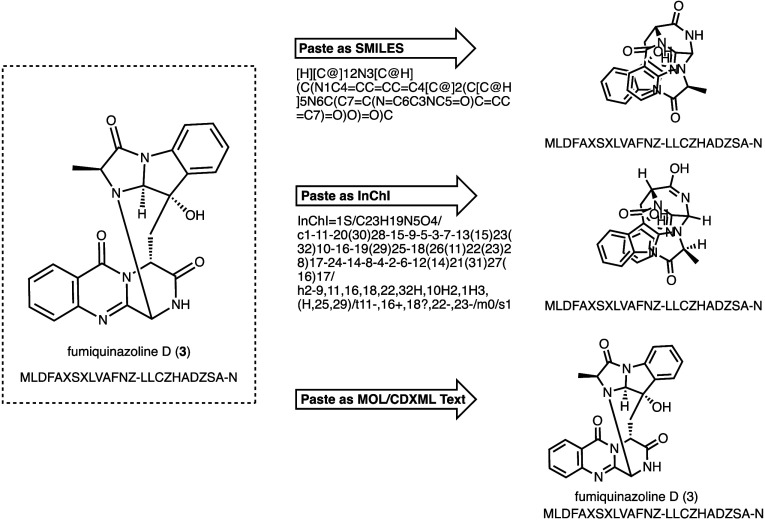
Structures regenerated from different machine-readable formats. The line notations SMILES and InChI can be decoded by chemical software, but they are subject to interpretation in terms of display angles and conventions/settings. As the MOLfile has the exact atom positions, this format allows faithful regeneration of the original perspective including text annotations. Regardless of the perspective, we can generate the InChIKey for each of these depictions and find that they all are the same hash and thus are all the same molecule. We fully recognize that the clutter in the visual representation in the top two regenerated structures is hard for a chemist’s eyes; however, it is a nonfactor to cheminformatic searches, analyses, and calculations. Rather, it is important that the structural information is correct. Whether the structure looks pleasing to the chemist’s eye (i.e., at the bottom/right) or is somewhat cluttered (as shown in the top/middle), those structures are identical.

Another option for storing and sharing machine-readable structure information is chemical table files, such as MOLfiles.^54^ Fundamentally, these are files recording the position of atoms in 3D space, and this file type may be more common to those working in computational chemistry or cheminformatics. The atom positions are *xyz*-coordinates (in Å) listed in an “atom block”, and the bonds are specified in the “bond block” part of the file, in addition to other information. These files allow the sharing of the exact structure without the need for any interpretation by the chemical drawing software, which guarantees the exact layout will be regenerated. Additionally, this is the only format capable of faithfully capturing atropisomers or axes of chirality. However, being a separate file mandates solutions for storage and sharing different from the line notations of SMILES and InChI.

Finally, the InChIKey descriptor allows for the verification and authentication of different versions of structural drawings. In contrast to SMILES, InChI, and MOLfiles, InChIKey is not a file format from which a structure can be regenerated. Rather, it is a fixed-length hash (an abstraction calculated by an algorithm^55^) of the InChI of a structure. As there is exactly one InChI per compound, there is also exactly one InChIKey. The first part of the InChIKey compresses information on how atoms are bonded in the “connectivity layer” into a string of 14 letters. The second part of InChIKey encapsulates the absolute configuration, charges, and other attributes into a string of 10 letters. A final letter describes the protonation of the molecule, where “N” indicates “neutral”, i.e., no proton-related ionization.^49^

As the InChIKey is produced by a freely available algorithm,^55^ anyone can calculate the InChIKey descriptor for a structure, always yielding the same exact InChIKey for the same molecule. This allows for verification and authentication of different versions of structural drawings, such as depictions from different perspectives/rotated structures (Figures 1 and 3). To do so, one can simply compare the InChIKey of a structure in question with the InChIKey of the reference structure (ideally published along with the original description of the structure of a new secondary metabolite; see our recommendation in the final section) to verify its fidelity. Crucially, the InChIKey is also a natural, universal interface connecting all chemical databases, which allows for fast querying for the presence of a compound.

In reality, there are a variety of different line notations and file formats for chemical structures that have been developed in the past.^56^ And with emerging new uses, new formats are being developed, such as SELFIES for machine learning chemistry.^57^ However, the ones suggested in this Perspective are the most used across existing databases, and they seem to be valid alternatives for the workflow of natural products chemistry and beyond.^16,58,59^

The utility of such identifiers is best illustrated through a real-world example, as shown in the next section.

## Example Case: Pyrichalasin H

The fungal secondary metabolite pyrichalasin H (**1**) offers a nice illustration of the presence and propagation of erroneous structures and how these could have been avoided if machine-readable structure representations were part and parcel of natural product chemistry publications. Pyrichalasin H (**1**) was first described by Nukina in 1987 ^47^ (Figure 1A) and reported again by Wang et al. when its BGC was discovered.^60^ While the drawing of **1** from the original publication (Figure 1A) is a bit different than ACS standards of 2025, this representation depicts the absolute configuration across nine stereogenic centers and two *E* double bonds; these were deduced based on comparisons to a related secondary metabolite, cytochalasin H (**2**), which had been determined earlier via chemical, spectroscopic, and X-ray crystallographic studies.^61^ Unfortunately, the structure of pyrichalasin H (**1**) is drawn differently across various large chemical databases. The PubChem version (Figure 1B) has taken the structure (**1**) and rotated it 180° along a vertical axis. While the designations of the double bonds in the macrocycle are still *E*, the configurations of the asymmetric centers are ambiguous. Accordingly, the InChI of this version lacks the layer designating the absolute configurations across nine stereogenic centers (highlighted in blue in Figure 1; note that “t” indicates “tetrahedral”) (/t18-,19+,24-,25-,26+,27-,28+,30-,31+/m0/s1). Likewise, the InChIKeys differ between the versions in panels A and B, specifically in the second part, hashing the configuration (i.e., starting with the letters PWS).

NPAtlas (Figure 1C) depicts the structure from the same perspective as Figure 1A and also has the *E* designation of the double bonds; however, it too does not give the configuration of the stereogenic centers, depicting the same changes compared to the original and thus the same InChI and InChIKey as Figure 1B. The fact that the perspectives of the molecule are rotated between panels B and C is due to different cheminformatic software interpreting these line notations. Interestingly, the version in COCONUT (Figure 1D) is almost the opposite of the NPAtlas version, where in this case, the perspective is identical to the original drawing (Figure 1A) and the stereogenic centers are defined, but the double bonds are both shown as *Z* (but annotated with question marks). Accordingly, in the InChI, the layer denoting the geometry of the double bonds “/b” (/b 9–7+,15–14+; highlighted in yellow in Figure 1) is missing. Additionally, the Figure 1D drawing breaks a rule of organic chemistry, namely, that the circled carbon atom should not have a down wedge stereodescriptor bond going both to it and away from it, simultaneously. If one draws the molecule this way in commonly used chemical drawing editors, such as ChemDraw, the program will indicate a structural error warning at that position.

The point we want to emphasize is that there is one secondary metabolite named pyrichalasin H (**1**), which is depicted in panel A. None of the other molecules shown in panels B, C, or D are currently described or known to exist, and they are not **1**. The consequence of having three different structures of what should be the same molecule is that if one were to calculate, for example, the molecular properties of pyrichalasin H (**1**), the results could differ based on which structure was used. Correct answers can be obtained only from analyses of the structure shown in Figure 1A.

If one could go back in time to when the structure of pyrichalasin H (**1**) was first published and include line notations for the structure and the InChIKey, then such errors could have been avoided. Furthermore, irrespective of how the drawing appears in various databases, one could quickly authenticate the validity of the structure by comparing the InChIKey. We fully recognize that those options were not available to Nukina^47^ in 1987, but they are available today, and if we as a community start using them, similar errors could be avoided in the future.

## Adding SMILES, InChI, MOLfiles, and InChIKey Descriptors Is Straightforward

To publish the structure of a new secondary metabolite, the structural drawing is already in a software of the author’s choice. Commonly used chemistry software makes it straightforward to automatically generate a molecule’s SMILES, InChI, MOLfile, and InChIKey descriptors. For example, in ChemDraw, all one needs to do is highlight the structure, Edit > Copy as > “SMILES”, “InChI”, “Mol Text”, or “InChIKey”. Free alternatives also exist (e.g., https://www.rcsb.org/chemical-sketch). For publishers, these small additions will not take up much article space; they could be included in the main text of the article or as a table in the Supporting Information, effectively showing the data for the structure, the SMILES, the InChI, the MOLfile, and the InChIKey in a tabular format.

We advocate for the adoption of these changes across all journals and articles describing new structures of secondary metabolites. Additionally, we encourage authors to adopt this practice now (and are doing so ourselves^19^) by including the machine-readable text descriptors for both new and known compounds in the Supporting Information of future manuscripts, ahead of formal editorial guidance. While we have centered our discussion around compounds derived from Nature, similar arguments could be made about the value of machine-readable structure representations for the synthetic, medicinal, and organometallic chemistry communities. We note that our argument aligns with the FAIR principles, championing easier access to research findings, and also greatly facilitates integration into automated, machine-readable data deposition and curation.^23^
